# Compacted Sewage Sludge as a Barrier for Tailings: The Heavy Metal Speciation and Total Organic Carbon Content in the Compacted Sludge Specimen

**DOI:** 10.1371/journal.pone.0100932

**Published:** 2014-06-30

**Authors:** Huyuan Zhang, Qing Zhang, Bo Yang, Jinfang Wang

**Affiliations:** 1 Key Laboratory of Mechanics on Disaster and Environment in Western China, Lanzhou University, Ministry of Education, Lanzhou, P. R. China; 2 College of Earth and Environmental Sciences, Lanzhou University, Lanzhou, P. R. China; 3 School of Chemistry and Environmental Science, Lanzhou City University, Lanzhou, P. R. China; University of Kentucky, United States of America

## Abstract

Acid mine drainage (AMD) was the main environmental problem facing the mining industry. For AMD had high heavy metals content and low pH, the compacted sewage sludge might be a barrier for tailings whose oxidation and weathering produced AMD, with its own carbon source, microorganism reduction ability and impermeability. To study the heavy metals environmental risk, under the simulate AMD, the deionized water (DW), and the pH 2.1 sulfuric acid water (SA) seepage conditions, respectively, the changes of the chemical speciation of heavy metals Cd, Cu, Fe, Ni, Zn and total organic carbon (TOC) content in the compacted sewage sludge were assessed in the different periods. The results indicated according to the distribution of heavy metals, the potential mobility was for Cd: 6.08 under AMD, 7.48 under SA, ∞ under DW; for Cu: 0.08 under AMD, 0.17 under SA, 0.59 under DW; for Fe: 0.15 under AMD, 0.22 under SA, 0.22 under DW; for Ni: 2.60 under AMD, 1.69 under SA, 1.67 under DW; and for Zn: 0.15 under AMD, 0.23 under SA and 0.21 under DW at the second checking time. TOC content firstly decreased from 67.62±0% to 66.29±0.35%, then increased to 67.74±0.65% under the AMD seepage while TOC decreased to 63.30±0.53%, then to 61.33±0.37% under the DW seepage, decreased to 63.86±0.41%, then to 63.28±0.49% under SA seepage. That indicated under the AMD seepage, the suitable microorganisms communities in the compacted sewage sludge were activated. And the heavy metals environmental risk of compacted sewage sludge was lower with AMD condition than with other two. So the compacted sewage sludge as a barrier for tailings was feasible as the aspect of environmental risk assessment.

## Introduction

Acid mine drainage (AMD) was the largest, and most testing, environmental problem facing the mining industry today in the world, which caused by oxidation and weathering process of sulfides mainly in the sulfidic mine waste-tailings, because of low pH and organic content, high heavy metals and sulfate content [1]. In case AMD production, established sulfidic mine waste control strategies included barriers (i.e. wet and dry covers, compacted clay liner or geomembrane liner for the tailings), selective handling and isolation, co-disposal and blending with other materials, addition of organic wastes, and bacterial inhibition [2]–[8]. However, most of the strategies were only to encapsulate or only to eliminate.

Nason [9] remediated some sulfidic mine tailings of Sweden in an 8-year pilot scale experiment using sewage sludge to evaluate its applicability as a sealing layer in a composite dry cover. And he found the limiting factor to the function of the sealing layer might be organic matter degradation in the sludge, for the aerobic and anaerobic degradation resulted in 85% loss of the organic matter over the 8-year. However, Zhang [10] [11] evaluated the feasibility of the compacted sewage sludge as the barrier for tailings using the batch experiment, hydraulic conductivity measurement and oxidation titration to test its impermeability, interdiction to the heavy metals and oxidation buffering capability (OBC). The results indicated the hydraulic conductivity of compacted sewage sludge was 3.0×10^−8^ to 8.0×10^−8^ cm s^−1^, which met the requirement of the landfill liner 1.0×10^−7^ cm s^−1^, and that the eliminative rate to the heavy metals Zn and Cd was 97.8% and 93.4% respectively. Modeling the OBC depleting in the AMD seepage indicated the 2-meter thick compacted sewage sludge liner could keep a strong reductive status after 38787 years running with 10-meter depth of AMD hydraulic head. That was, if we thickened the compacted sewage sludge liner, Nason's [9] problem might be accomplished. Because of the microorganisms activities to eliminate the heavy metals, i.e. anaerobic sulfate reduction etc. in the compacted sewage sludge and its impermeability-the main property of the liner, the compacted sewage sludge, with double functions-both to encapsulate and to eliminate at the same time, could be the innovative liner for the tailings.

Components of the sewage sludge were very complex, however, containing a lot of organic material, heavy metals, salts, and radionuclide, etc. Emissions might cause serious pollution to the environment, especially the groundwater, if not treated properly [12], [13]. If the compacted sewage sludge functioned as the liner for the sulfidic mine tailings, both the introduced high concentration heavy metals in AMD seepage deposition and its original heavy metals amount might affect the environment together [14]. Total heavy metal content in the sewage sludge didn't well reveal its biological toxicity, its chemical activity and remigration in the environment. The environmental behavior and biological effects of heavy metals was not simply determined by their total content, but by the chemical speciation [15]–[17]. Different heavy metal speciation in the sewage sludge might have different bioavailability. Among the researches of the heavy metal speciation, Tessier [18] Sequential Extraction Procedure had been widely applied. This method separated the speciation of heavy metals in the nature into 5 fractions: Exchangeable Fraction, Carbonate Fraction, Fe-Mn-Oxide Fraction, Organic Fraction, and Residual Fraction. The former three kinds of fractions had poorer stability, easier to affect the organisms; and the latter two had the stronger stability, not easy to release into the environment [19].

Under the stress of low pH and high content of heavy metals, in the compacted sewage sludge being both the microorganisms species sources and carbon sources, the microorganism population would decrease and microorganism community and structure would change, so the microorganism biomass would decrease accordingly [20]. Together with the depletion of organic matters, total organic content (TOC) would decrease in the sewage sludge. So after a certain interval, to test whether the suitable microorganism populations adapt the stress and make the bioreduction, TOC was a good assessment index to show this change.

This study split the compacted sewage sludge specimens, during the different seepage periods under the simulate AMD permeant liquids, to assess the change of the chemical speciation of heavy metals and TOC content, and to elucidate the heavy metals migration potential, bioavailability, so as to provide useful references for the feasibility of the compacted sewage sludge barrier in the aspect of environmental risk assessment.

## Materials and Methods

### Material and experimental design

The sewage sludge used in this study was sampled from Yanerwan municipal sewage water treatment plant in Lanzhou, Gansu province, China. The sludge was air-dried, ground and passed through 0.5-mm sieve for tests. The chemical and geotechnical characteristic of the sewage sludge were presented in Tab. 1.

**Table 1:** The chemical and geotechnical characteristic of sewage sludge.

Characteristic	Values
Water content (%)	
Natural	75.03
Air-dried	8.21
pH	
Natural	6.47
Air-dried	6.35
Liquid limit (%)	207.14
Plastic limit (%)	43.64
Maximum dry density (g cm^−3^)	0.57
Optimum moisture content (%)	80.46
Conductivity (µS cm^−1^)	3172
TOC (%)	67.62
TN (%)	5.62
A-P (%)	2.05
Total Cd (mg kg^−1^)	9.10
Total Cu (mg kg^−1^)	106.84
Total Fe (mg kg^−1^)	578.54
Total Ni (mg kg^−1^)	43.21
Total Zn (mg kg^−1^)	503.58

Specimens for the seepage experiment were prepared by adding deionized water to the air-dried sewage sludge to achieve the water content 2% wet of the optimum moisture content. Afterward, the moistened sewage sludge was sealed in plastic bags and allowed to hydrate for 24 h. Then, the sewage sludge was compacted following a standard Proctor compaction method (ASTM D 698) in a cylindrical steel mold that was 102 mm in diameter and 116 mm in height. Once the compaction was completed, the specimen was extruded and trimmed to the desired dimensions (102 mm in diameter and 40 mm in height). Subsequently, the specimen was placed between filter papers and porous stones, then sheathed in a rubber membrane and finally set in a flexible-wall permeameter.

Three different solutions were used to simulate acid pore water and used as the permeant liquid: the synthetic AMD, the deionized water (DW), and the pH 2.1 sulfuric acid water (SA). The synthetic AMD contained Fe (1,500 mg L^−1^), Zn (350 mg L^−1^), Cu(35 mg L^−1^), Ni(182 mg L^−1^), Cd (100 mg L^−1^), SO_4_ (4,500 mg L^−1^), and Ca (200 mg L^−1^), and was prepared with DW water using FeSO_4_·H_2_O, ZnSO_4_·7H_2_O, CuSO_4_·5H_2_O, NiSO_4_·6H_2_O,CdSO_4_ and CaCl_2_. In addition, H_2_SO_4_ was added in the synthetic AMD to achieve pH 2.1. The pH 2.1 sulfuric acid water (prepared using DW water and H_2_SO_4_) was used to distinguish the effects of metals and acidity on sludge specimens. DW water was used as a control. The composition of the synthetic AMD was selected by reviewing the 12 AMD for the metallic mine wastes according to the literature [21], [22].

**Figure 1:**
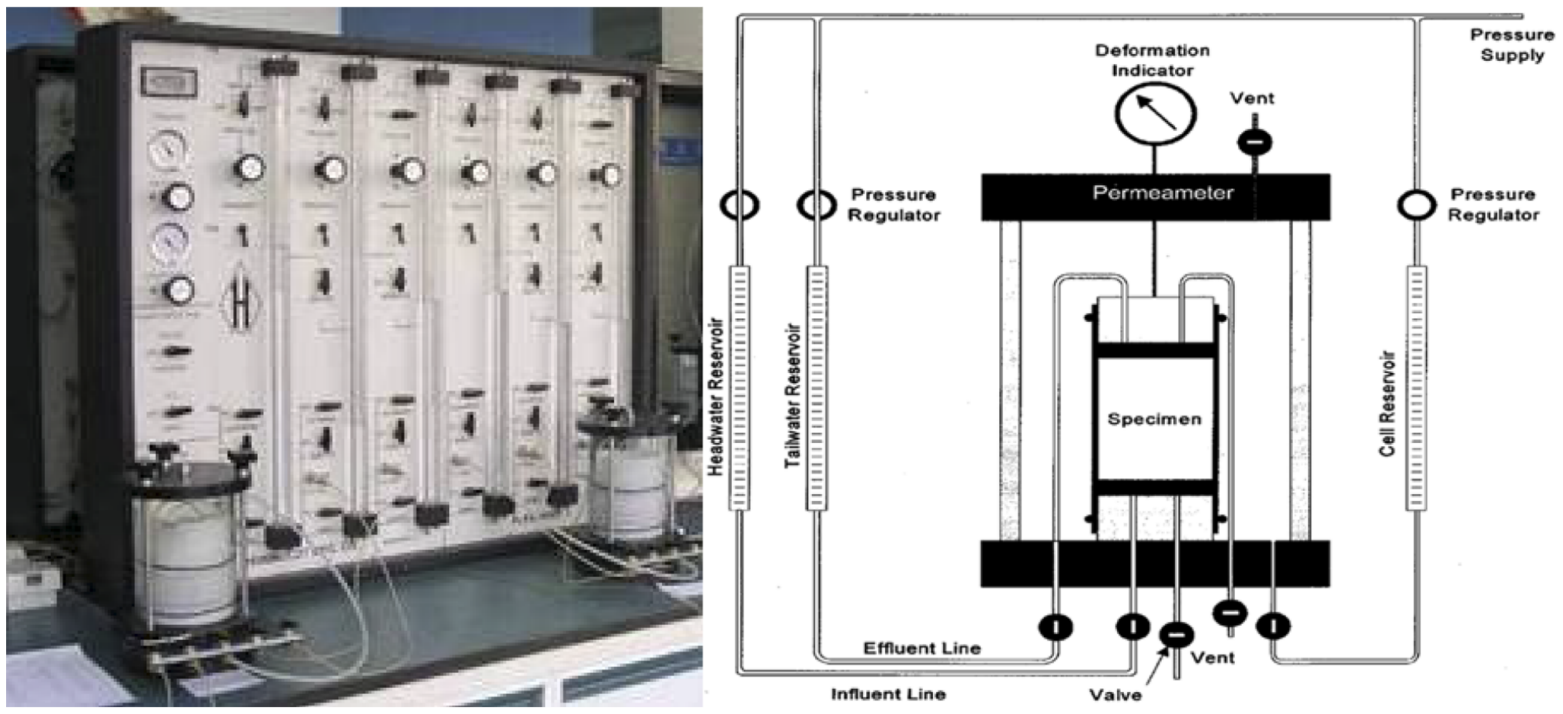
Schematic diagram of flexible-wall permeameter.

The hydraulic conductivity of the compacted sewage sludge was determined using a flexible-wall permeameter according to the ASTM D 5084 standard with the falling head method (see Fig.1). A confining pressure of 200 kPa was applied during the test following the recommendation of Kamon [23]. The readings of the influent at certain time intervals were recorded to calculate the hydraulic conductivities of the specimen according to Darcy's law.

### Sampling and analyses

The hydraulic conductivity was 7.8×10^−10^ cm s^−1^ with DW seepage condition, 7.6×10^−10^ cm s^−1^ with SA seepage condition and 6.8×10^−10^ cm s^−1^ with AMD seepage condition respectively at the first day and then decreased from the baseline value of 3.7×10^−10^ to 3.5×10^−11^ cm s^−1^ which was nearly impermeable. At the time of the 41st day (T1) and the 75th day (T2) respectively, the cells of the flexible-wall permeameter were opened and the sludge specimens were taken out to be air-dried, ground, passed through 0.5-mm sieve for the TOC and the speciation of Cd, Cu, Fe, Ni, Zn tests.

TOC was determined by TOC analysismeter (LiquiTOC/TNb, ELEMENTAR, Germany).

The heavy metals speciation of Cd, Cu, Fe, Ni, Zn were tested as per Tessier (1979) [18] using Atomic Absorption Spectrometer (SPSIC 4530F, China).

The chemical characteristic determination of compacted sewage sludge as per the reference [24]. The geotechnical characteristic determination as per the standard Proctor compaction method (ASTM D698).

## Results and Discussion

### The distribution and the concentration of heavy metals

**Figure 2:**
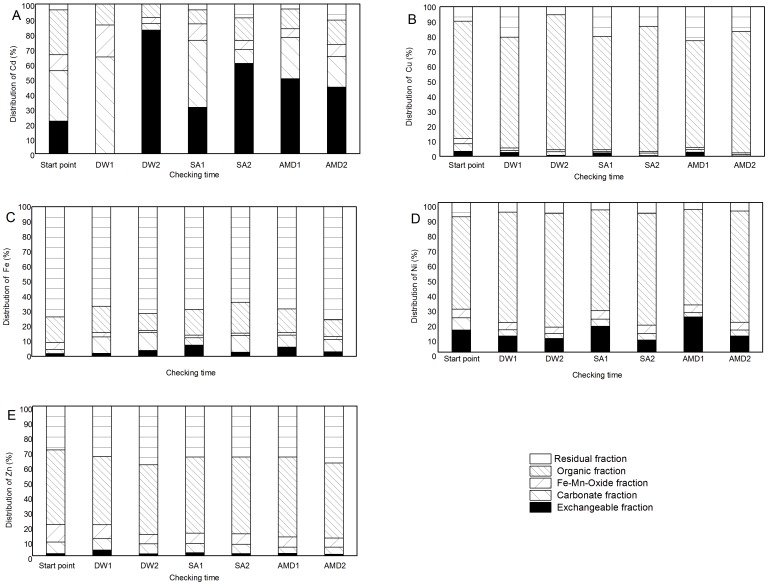
The distribution of heavy metals: Cd (A), Cu (B), Fe (C), Ni (D), Zn (E) in the compacted sludge specimens, with the deionized water (DW), pH 2.1 sulfuric acid water (SA), or the synthetic AMD as the permeant liquid at start point (SP), time1(T1) and time2(T2).

The distribution of heavy metals Cd, Cu, Fe, Ni, Zn in different sludge specimen at T1 and T2 was analyzed respectively by sequential extraction experiments. The results were shown in Tab.2-Tab.6 and Fig.2.

In Tab. 2 and Fig.2(A), at the start of the flexible-wall permeameter experiment with the deionized water (DW) as the permeant liquid, in the compacted sludge specimen, the cadmium was distributed mainly in the carbonate fraction (33.64%) and in the organic fraction (30.16%) as the concentration tendency was 3.06 mg kg^−1^ and 2.75 mg kg^−1^ respectively. The residual fraction of the cadmium was the least, only 3.79% as the concentration tendency was 0.34 mg kg^−1^. At the T1 of the flexible-wall permeameter experiment, the heavy metal of Cd was distributed mainly in the carbonate fraction (64.72%) as the concentration of 1.47 mg kg^−1^ while the exchangeable fraction decreased to 0% and the residual fraction also decreased to 0%. At T2 of the experiment, the exchangeable fraction of Cd increased to 82.66% as the concentration tendency 13.35 mg kg^−1^ while the carbonate fraction of Cd decreased to 4.45% as the concentration tendency of 0.72 mg kg^−1^ and the organic fraction of Cd decreased from 14.03% at the T1 to 8.95% as the concentration tendency of 1.45 mg kg^−1^. The residual fraction of Cd still remained 0%. In this experiment, the Fe-Mn-Oxide fraction of Cd changed from 10.60% as the concentration tendency of 0.96 mg kg^−1^ at the start to 21.25% as the concentration tendency of 0.48 mg kg^−1^ at the T1 to 3.94% as the concentration tendency of 0.64 mg kg^−1^ at the T2.

**Table 2:** The concentration tendency of Cd in the compacted sludge specimens, with the deionized water (DW), pH 2.1 sulfuric acid water (SA), or the synthetic AMD as the permeant liquid at the start point (SP), time1(T1) and time2(T2).

Concentration tendency of Cd (mg kg^−1^)	Different checking time						
	SP	DW1	DW2	SA1	SA2	AMD1	AMD2
**Exchangeable fraction**	1.98	0.00	13.35	3.39	7.29	2.04	3.42
**Carbonate fraction**	3.06	1.47	0.72	31.01	1.09	1.12	1.56
**Fe-Mn-Oxide fraction**	0.96	0.48	0.64	44.75	0.75	0.42	0.61
**Organic fraction**	2.75	0.32	1.45	11.01	1.81	0.54	1.25
**Residual fraction**	0.34	0.00	0.00	9.41	1.12	0.13	0.82

As shown in Tab.2 and Fig.2(A), with the pH2.1 sulfuric acid water (SA) as the permeant liquid, the exchangeable fraction of Cd changed from 21.81% at the start to 31.01% at the T1 then to 60.47% at the T2 as the concentration tendency of 1.98 mg kg^−1^, 3.39 mg kg^−1^and 7.29 mg kg^−1^ respectively. The carbonate fraction of Cd changed from 33.64% at the start to 44.75% at the T1, then to 9.01% as the concentration tendency of 3.06 mg kg^−1^, 31.01 mg kg^−1^, 1.09 mg kg^−1^ respectively. The Fe-Mn-oxide fraction of Cd changed from 10.60% at the start to 11.01% at the T1 to 6.18% at the T2 as the concentration tendency of 0.96 mg kg^−1^, 44.75 mg kg^−1^ and 0.75 mg kg^−1^ respectively. The organic fraction of Cd changed from 30.16% at the start to 9.41% at the T1, then to 15.04%, as the concentration tendency of 2.75 mg kg^−1^, 11.01 mg kg^−1^, 1.81 mg kg^−1^. The residual fraction of Cd changed from 3.79% at the start to 3.82% at the T1, then to 9.29%, as the concentration tendency of 0.34 mg kg^−1^, 9.41 mg kg^−1^, and 1.12 mg kg^−1^.

Also shown in Tab.2 and Fig.2(A), with the synthetic AMD as the permeant liquid, at the T1 and the T2 of the experiment, the cadmium in the sludge specimen was mainly distributed in the exchangeable fraction (T1:50.19%, 2.04 mg kg^−1^; T2: 44.59%, 3.42 mg kg^−1^) while at the start, Cd was mainly distributed in carbonate fraction (33.64%, 3.06 mg kg^−1^).

As shown in Tab.3 and Fig.2(B), at the T1 and T2 of the experiment, the copper mainly distributed in the organic fraction. With the DW water as the permeant liquid, the organic fraction changed from 78.41% (83.78 mg kg^−1^) at the start to 74.12% (167.70 mg kg^−1^) at the T1,then 90.26% (162.78 mg kg^−1^).With the sulfuric acid water as the permeant liquid, the organic fraction changed from 75.70% (167.50 mg kg^−1^) at the T1 to 83.69% (140.76 mg kg^−1^) at the T2. With the AMD as the permeant liquid, the organic fraction changed from 71.18% (116.51 mg kg^−1^) at the T1 to 81.09% (161.03 mg kg^−1^) at the T2.

**Table 3:** The concentration tendency of Cu in the compacted sludge specimens, with the deionized water (DW), pH 2.1 sulfuric acid water (SA), or the synthetic AMD as the permeant liquid at the start point (SP), time1(T1) and time2(T2).

Concentration tendency of Cu (mg kg^−1^)	Different checking time						
	SP	DW1	DW2	SA1	SA2	AMD1	AMD2
**Exchangeable fraction**	3.50	5.84	1.19	5.25	1.00	4.69	0.38
**Carbonate fraction**	5.50	2.94	4.50	1.81	2.69	2.88	2.19
**Fe-Mn-Oxide fraction**	3.75	3.75	2.19	2.73	1.80	2.34	1.95
**Organic fraction**	83.78	167.70	162.78	167.50	140.76	116.51	161.03
**Residual fraction**	10.31	46.02	9.69	43.98	21.95	37.27	33.05

As shown in Tab.4 and Fig.2(C), the iron mainly distributed in the residual fraction. With the DW water as the permeant liquid, the residue fraction changed from 73.53% (425.39 mg kg^−1^) at the start to 66.50% (432.99 mg kg^−1^) at T1, finally 71.23% (433.21 mg kg^−1^) at the T2. With the sulfuric acid water (SA) as the permeant liquid, the residue fraction changed from 68.48% (437.20 mg kg^−1^) at the T1 to 63.73% (419.52 mg kg^−1^) at the T2.With the AMD as the permeant liquid, the residual fraction of Fe changed from 68.21% (436.60 mg kg^−1^) at the T1 to 75.55% (425.17 mg kg^−1^) at the T2.

**Table 4:** The concentration tendency of Fe in the compacted sludge specimens, with the deionized water (DW), pH 2.1 sulfuric acid water (SA), or the synthetic AMD as the permeant liquid at the start point (SP), time1(T1) and time2(T2).

Concentration tendency of Fe (mg kg^−1^)	Different checking time						
	SP	DW1	DW2	SA1	SA2	AMD1	AMD2
**Exchangeable fraction**	12.09	14.24	24.46	48.52	19.21	40.56	17.78
**Carbonate fraction**	15.65	71.52	72.08	31.26	74.01	50.57	46.49
**Fe-Mn-Oxide fraction**	26.33	17.75	9.5	11.17	9.58	11.33	11.17
**Organic fraction**	99.07	114.64	68.95	110.30	135.94	101.03	62.14
**Residual fraction**	425.39	432.99	433.21	437.20	419.52	436.60	425.17

As shown in Tab.5 and Fig.2(D), the heavy metal of nickel mainly distributed in the organic fraction. The organic fraction of Ni was 61.72% (26.67 mg kg^−1^) at the start, 73.62% (59.58 mg kg^−1^) at the T1, then 75.72% (55.00 mg kg^−1^) at the T2 with the DW as the permeant liquid. With the sulfuric acid water (SA) as the permeant liquid, the organic fraction of Ni changed from 67.35% ((57.08 mg kg^−1^) at the T1 to 74.56% (47.08 mg kg^−1^) at the T2 while that of Ni changed from 63.80% (61.04 mg kg^−1^) at the T1 to 74.33% (65.21 mg kg^−1^) at the T2 with AMD as the permeant liquid.

**Table 5:** The concentration tendency of Ni in the compacted sludge specimens, with the deionized water (DW), pH 2.1 sulfuric acid water (SA), or the synthetic AMD as the permeant liquid at the start point (SP), time1(T1) and time2(T2).

Concentration tendency of Ni (mg kg^−1^)	Different checking time						
	SP	DW1	DW2	SA1	SA2	AMD1	AMD2
**Exchangeable fraction**	6.39	8.71	6.68	14.61	5.09	22.50	9.39
**Carbonate fraction**	3.53	3.40	2.38	4.15	2.87	2.65	3.62
**Fe-Mn-Oxide fraction**	2.50	4.03	3.13	4.72	3.40	5.00	4.51
**Organic fraction**	26.67	59.58	55.00	57.08	47.08	61.04	65.21
**Residual fraction**	4.12	5.22	5.44	4.19	4.71	4.49	5.00

As shown in Tab.6 and Fig.2(E), the heavy metal of zinc mainly distributed in the residual fraction and the organic fraction. With the DW as the permeant liquid, the residual fraction of Zn changed from 29.09% (146.52 mg kg^−1^) at the start to 33.63% (195.90 mg kg^−1^), then to 39.07% (214.32 mg kg^−1^) while the organic fraction of Zn changed from 49.95% (251.54 mg kg^−1^) at the start to 45.58% (265.51 mg kg^−1^), then to 46.71% (256.19 mg kg^−1^). With the SA as the permeant liquid, the residual fraction of Zn changed from 33.88% (173.65 mg kg^−1^) at the T1 to 33.92% (163.83 mg kg^−1^) while the organic fraction of Zn changed from 51.04% (261.57 mg kg^−1^) to 51.32% (247.89 mg kg^−1^) at the T2. With the AMD as the permeant liquid, the residual fraction of Zn changed from33.87% (167.24 mg kg^−1^) at the T1 to 37.94% (190.18 mg kg^−1^) at the T2 while the organic fraction of Zn changed from 53.53% (264.30 mg kg^−1^) at the T1 to 50.20% (251.63 mg kg^−1^).

**Table 6:** The concentration tendency of Zn in the compacted sludge specimens, with the deionized water (DW), pH 2.1 sulfuric acid water (SA), or the synthetic AMD as the permeant liquid at the start point (SP), time1(T1) and time2(T2).

Concentration tendency of Zn (mg kg^−1^)	Different checking time						
	SP	DW1	DW2	SA1	SA2	AMD1	AMD2
**Exchangeable fraction**	7.17	22.90	7.46	11.03	7.13	8.07	5.45
**Carbonate fraction**	38.79	43.55	36.90	31.62	29.87	20.92	24.01
**Fe-Mn-Oxide fraction**	59.57	54.70	33.61	34.65	34.32	33.19	30.02
**Organic fraction**	251.54	265.50	256.19	261.57	247.89	264.30	251.63
**Residual fraction**	146.52	195.90	214.32	173.65	163.83	167.24	190.18

### The TOC content of the compacted sludge specimen

**Figure 3:**
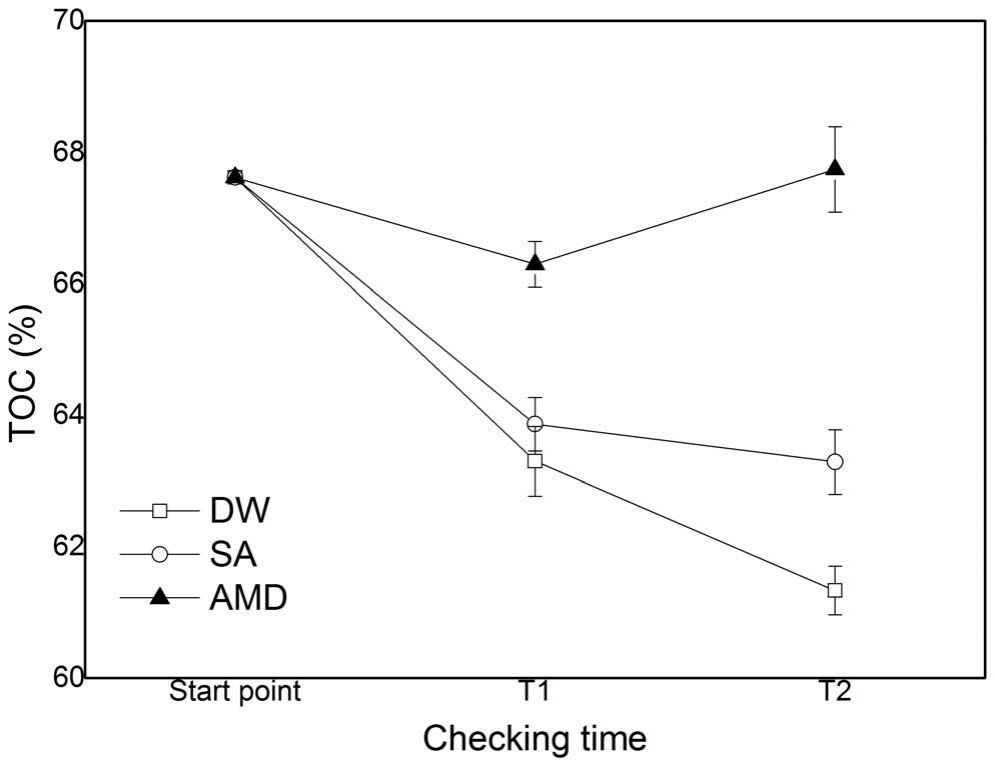
The TOC content(%) (mean ±SD) at the different checking time in the compacted sludge specimens with the deionized water (DW), pH 2.1 sulfuric acid water(SA) or the synthetic AMD as the permeant liquid respectively.

As shown in Fig.3, with the DW as the permeant liquid, the TOC in the compacted sludge specimen changed from 67.62±0% at the start to 63.30±0.53% at the T1, then to 61.33±0.37% at the T2. With the SA as the permeant liquid, the TOC content changed from 67.62±0% at the start, to 63.86±0.41% at the T1, then to 63.28±0.49% at the T2. With the AMD as the permeant liquid, the TOC content in the compacted sludge specimen decreased from 67.62%±0% at the start to 66.29%±0.35% at the T1, finally increased to 67.74%±0.65%. The TOC downward trend slowed.

There was a significant connection between the heavy metals speciation and its bioavailability and environmental effects. The exchangeable fraction and the carbonate fraction of the heavy metals had the most labile bond to the wastes, therefore, they were the most dangerous and bio-available for the environment. The Fe-Mn oxides fraction could be released if conditions changed from oxic to anoxic state. The organic fraction was referred to metals bound to sulfides and organic matter, which might be released under oxidizing conditions but relatively stable. The residual fraction strongly associated with crystalline structures of minerals, hence, the most stable, which were therefore unlikely to be released from the compacted sludge specimens [25]. According to Elliot et al [26], the ratio between (Exchangeable fraction + Carbonate fraction) and Residual fraction was regarded as the criterion to the potential mobility of the heavy metals. The higher the ratio meant the stronger the potential mobility of the heavy metals.

As for Cd, although the percentage of the exchangeable fraction and the carbonate fraction exceeded 82% with DW permeant liquid at T2, it decreased to 69% and 64% with SA and AMD permeant liquid, which meant the ability to fix Cd of compacted sludge specimen be get raised with the latter seepage conditions. See Tab.7, at T2, Cd potential mobility was the lowest(6.08) with AMD permeant liquid compared with SA, 7.48 and DW, ∞. It also meant the compacted sewage sludge with introduced Cd content, under pH 2.1 condition had less environmental risk.

**Table 7:** The heavy metals potential mobility in the compacted sewage sludge in the different periods (start point, time1 and time2) with different permeant liquids.

Potential Mobility (EXC+CAR)/RES																
Heavy metals		Cd			Cu			Fe			Ni			Zn		
Permeant liquid		DW	SA	AMD	DW	SA	AMD	DW	SA	AMD	DW	SA	AMD	DW	SA	AMD
**Checking time**	SP	14.63	14.63	14.63	0.87	0.87	0.87	0.07	0.07	0.07	2.41	2.41	2.41	0.31	0.31	0.31
	T1	∞	3.66	24.43	0.19	0.16	0.20	0.20	0.18	0.21	2.32	4.48	5.61	0.34	0.25	0.17
	T2	∞	7.48	6.08	0.59	0.17	0.08	0.22	0.22	0.15	1.67	1.69	2.60	0.21	0.23	0.15

As for Cu, at T1 and T2, the percentage of the organic fraction and the residual fraction both exceeded 93%, but also the percentage of T2>T1.Which meant with time, the result to fix Cu became better. In its distribution, the organic fraction exceeded 71%, and the changing curve conformed to the TOC changing. The patterns of Ni and Zn were like Cu, although the organic fraction percentage of those were lower than Cu. It was because that the organic matters might combine the heavy metals to conform the organic-metal complexes, which stabilized the heavy metals. And the content of the organic matters would increase or decrease synchronously with the heavy metals [27]. See Tab.7, the Cu potential mobility with AMD permeant liquid (0.08) was the lowest among that with DW (0.59) and SA (0.17) at the T2.

As for Fe, over 65% fractions were distributed in the residual fraction whether with DW or with SA, AMD as the permeant liquid. In addition, the percentage of the exchangeable fraction and the carbonate fraction under SA and AMD seepage was lower than that under DW. That was, see Tab.7, the Fe potential mobility at T2 with AMD permeant liquid was the lowest, 0.15. Which meant the SA and AMD decreased the environmental risk of the compacted sludge specimens. And so was Zn. With AMD permeant liquid, potential mobility was only 0.15.

As for Ni, the only exception, at T2, with AMD permeant liquid, the potential mobility was the highest, 2.60.

All for heavy metals, Cd, Cu, Fe, Ni, Zn, the mobility were lower at T2 than that at the start point.

As the degradation product accumulation, biodegradable material reduction, the original microorganisms community growth was inhibited, and TOC content decreased continuously before T1 with all 3 permeant liquid. Till T2, the suitable microbial populations began to proliferate, however, causing bacteria ingredient such as fat and protein content increase, so the TOC content slightly increased at T2. With the function of geochemical and microorganism reduction, the potential mobility of heavy metals decreased. And with AMD permeant liquid, TOC downward trend was slowed.

## Conclusions

The compacted sewage sludge barrier might utilize its own carbon source and microorganism source to fix the heavy metals under the simulate AMD seepage condition better than the other two seepage conditions. Among the potential mobility of Cd, Cu, Fe, Zn assessment, the combination between compacted sewage sludge and simulate AMD was the lowest, that was, the environmental risk was lower than the other combinations.

The compacted sewage sludge as a barrier for the tailings might be feasible as the aspect of environmental risk assessment.
